# The role of Bio-C Plus in reducing the detrimental impacts of heat stress on the production and reproduction of pregnant rabbits and their young from birth to weaning

**DOI:** 10.1007/s11250-025-04374-y

**Published:** 2025-04-05

**Authors:** Alsaied Alnaimy Habeeb, Anhar I. A. Elhanafy, Ahmed K. Sharaf, Mostafa A. A. Atta

**Affiliations:** Biological Applications Department, Egyptian Atomic Energy Authority, Nuclear Research Center, Radioisotopes Applications Division, P.O. 13759, Cairo, Egypt

**Keywords:** Rabbits, Thermal stress, Bio-C plus, Milk yield, Reproductive efficiency

## Abstract

This research aimed to evaluate the detrimental impacts of heat stress and Bio-C Plus, an innovative technique to mitigate summertime heat stress, on the production and reproduction of pregnant rabbits and the growth of their offspring from birth to weaning. In two tests, a total of sixty adult, virgin New Zealand rabbits were employed in the current investigation. The first experiment was completed on twenty female pregnant rabbits in the winter season. Another experiment was conducted on forty rabbits, which was approved during the sweltering heat. The animals were split into two groups for the summer trial, with twenty individuals each. The first was kept in an additive-free environment, and the second added 1 g of Bio-C plus to every liter of drinking water for the doe rabbits. The findings demonstrated that pregnant rabbits subjected to extreme heat stress during the hot summer months experienced a considerable loss in body weight and feed intake and a considerable drop in conception rate. The summer heat stress has a detrimental effect on the litter weight and size of pregnant rabbits both at birth and at weaning. When Bio-C is added to drinking water, the effects of this stress are lessened.

## Introduction

The ideal weather for rabbits would be between 13 and 20 °C in the air, 5 to 18 km/h in the wind, 55 to 65% relative humidity, and moderate amounts of sunshine. Rabbits experience heat stress when these environmental elements change, which makes it difficult for their bodies to expel extra heat. As a result of climate change, rabbits will be subjected to extreme weather stress for nearly half the year. When rabbits are exposed to heat stress, their bodies go through a series of dramatic changes in function (Habeeb et al. 2023). These include imbalances in blood metabolites, enzymatic activity, water, protein, energy, and mineral balances, hormonal secretions and impaired reproductive and productive performance (Habeeb 2019). Climate change has posed a significant risk to livestock production in the recent past, particularly concerning the anticipated increase in global surface temperature. Due to their condensed coat and seldom-functioning sweat glands, which significantly reduce heat dissipation, rabbits are more vulnerable to heat stress situations (Marai et al. 2002). Rabbits are highly susceptible to heat stress, particularly during pregnancy, which is thought to harm the majority of their physiological and reproductive characteristics as well as their fertility (Kalaba 2012; Sabah et al. 2016). Furthermore, prolonged exposure to heat stress increases the amount of free radicals in the animal, which can cause oxidative stress (Kumar et al. 2011). According to Tremellen (2008), oxidative stress arises when the body's natural antioxidant defense system is outmatched in producing potentially harmful reactive oxygen species. Elevated oxidative stress in females raises the likelihood of spontaneous abortion along with other variables such as litter performance, animal welfare, and health status, which include decreased lifespan, milk supply, and reproductive success (Zhao et al. 2011). Rabbits, on the other hand, are homoeothermic animals that can control their body's heat generation and intake through morphological, biochemical, behavioral, and physical mechanisms, allowing them to maintain a somewhat normal body temperature (Habeeb 2020). This study aimed to evaluate the impact of heat stress and a new supplement known as Bio-C Plus on the rate of conception, the efficiency of reproduction, the quantity of milk produced, and different oxidative stress markers in pregnant rabbits, as well as the growth of their young from birth to weaning.

## Material and methods

### Experimental location

The experimental setup was the rabbitry of the Experimental Farms Project, Radioisotopes Application Division, Nuclear Research Centre, Atomic Energy Authority, Inshas, Egypt (latitude 31º 12' N to 22º 2' N, longitude 25º 53' E to 35º 53' E), throughout the winter and summer seasons.

### Determination of heat stress thresholds for the Temperature-Humidity Index (THI)

An automated thermo-hygrometer was used once a week to record the indoor ambient temperature and relative humidity inside the rabbit building to determine the temperature humidity index (THI). The formula used to compute the THI was THI = T—[(0.31–0.31 × RH) (T–14.4)], where T is the ambient temperature and RH is the relative humidity expressed as a percentage of 100. The THI values were contrasted with the classification as indicated below: According to Marai et al. (2001), < 27.8 indicates little heat stress, 27.8–28.9 indicates moderate heat stress, 28.9–30.0 indicates severe heat stress, and beyond 30 indicates very severe heat stress.

### Animals, feeding and housing

The experimental female rabbits were fed identical feed from the El-Mostafa Establishment, which manufactures feed for the Sharkia state of Egypt, for the duration of the study. The contents of the marketable diet consist of 40% clover hay, 25% wheat bran, 15% yellow maize, 10% soybeans, molasses, 5% bone meal, 1% calcium carbonate, 1% sodium chloride, 0.5 percent vitamins and minerals premix, and 0.5 percent DL-methionine. According to the marketable food's chemical analysis, the dry matter percentages of ash, ether extract, nitrogen-free extract, crude protein, and crude fiber are 18.5, 12.5, 3.5, 56, and 9.5%, respectively. There is a further 2600 kcal/kg DM of digestible energy. Every female rabbit was kept in a galvanized metal hutch measuring 80 cm by 60 cm by 60 cm and positioned 100 cm above the floor. Hutches were cleaned, sanitized, and let dry for a week before they were placed in their new homes. Each cage also has a feeder for feeding the edible feed and a nipple for drinking. Water and feed were provided *adlib* to each experimental group. Pens are cleaned daily, and their excrement and pee are discharged. The rabbits were protected from Clostridium enterotoxaemia bloat before the experiment.

### Experimental design

Two experiments with a total of sixty virgin adult female New Zealand White (NZW) rabbits were conducted for this investigation. The first one was done in the winter on twenty female rabbits during January and February of 2023. The animals were subjected to mild winter conditions, with ambient temperature (AT) and relative humidity (RH %) recording of 20.86 °C and 63.18%, respectively. Forty rabbits participated in the second experiment, which was carried out in July and August of 2023 during the sweltering heat, with respective AT and RH% values of 30.90 °C and 81.63%. The animals were split into two groups for the summer trial, with twenty individuals each. The first group was kept in an additive-free environment, and the second group was given 1 g of Bio-C plus per liter of drinking water. This rate complies with the recommendations for small animals. Mega-Biotic, located in 10th of Ramadan city, Sharkia state, Egypt, is the manufacturer of Bio-C Plus. Ascorbic acid (180.0 g), vitamin E (30.0 g), zinc sulphate (30.0 g), calcium gluconate (100.0 g), sodium chloride (150.0 g), potassium chloride (75.0 g), citric acid (200.0 g), and up to 1 kg of dextrose are all included in each kilogram of Bio-C Plus. The Bio-C Plus material dissolves fully in the water. The experimental doe rabbits were kept in a galvanized metal hutch for a week before each experiment, to allow for acclimatization before being introduced to the male for mating. The two tests' animal subjects were housed in identical administrative and sanitary settings. Every week, feed consumption and live body weight were recorded. The live body weight of each pregnant rabbit was determined on a weekly basis. Every week at 14.0 h, each doe's individual respiratory rate and ear temperature were recorded. When the rabbit was at rest, the number of breaths it took was counted for one minute, measuring the rise in its chest. This allowed researchers to calculate the rabbit's respiratory rate. By putting the thermometer in direct contact with the middle of the auricle, the temperature of the ear was determined.

### Blood samples and analysis

Every week, until two days before the kidding process, blood samples were taken from each doe to measure γ-globulin, progesterone hormone, and several oxidative stress markers. Following clotting, a clean test tube was used to hold the blood sample that was taken out of the ear vein by puncturing it. It was then centrifuged for 25 min at a speed of 3500 rpm. Before analysis, the serum was separated and refrigerated at −20 °C. As the manufacturer directs, perform a progesterone assay utilizing the direct radioimmunoassay method (RIA) on a ready-made antibody-coated tube kit (Diagnosis Systems Laboratories, Texas, USA). The hormone tracer was labelled with iodine-125 (I^125^). Superoxide dismutase (SOD) and glutathione peroxidase (GPX) enzymatic activity in serum was measured using spectrophotometer techniques, and commercial reagent kits were supplied by Bio-diagnostic, Cairo, Egypt.

### Estimating the milk output, conception rate, and reproductive efficiency

Ten days following mating, rabbits were manually checked for pregnancy. The thumb and forefinger were used to palpate the doe's lower belly, looking for marble-sized nodules. The rate of conception was noted for every group when the pregnancy was verified. The number of does conceive divided by the total number of does mate and multiplied by 100 was used to determine the conception rate. Following kidding, estimates were made of the litter size at delivery (which is the total number of kits delivered, including stillbirths), the weight of each litter at weaning, and the weight of the bunnies during the breastfeeding phase. The proportion and quantity of deaths were also noted. The body weight of the newborn, as well as the weekly and post-weaning weights, was also measured. Mortality rate during suckling period, either number or percentage, was also measured. The daily milk yield (g) per doe was measured once weekly during the suckling period using the weight bunnies of each doe before and after the completion of suckling at midday of the second day from isolation and the isolation of all kits out of their moms at midday.

### Statistical analysis

The SPSS (2012) version 19 process was used to statistically analyse the data in accordance with this model: Y_ij_ is equal to µ + T_i_ + e_ij_, where e_ij_ is the residual error, µ is the overall mean, and T_i_ is the fixed impact of treatments (1 being winter, 2 being summer without treatment, and 3 being summer with Bio-C Plus). The substantial discrepancies between means were examined using Duncan's innovative multiple ranges test (Duncan 1955). The Chi-square test was used to assess statistical differences between treatments in the likelihood of the conception rate and death rate percentages. Multiple Z-tests were then used to compare matching proportions and identify significant findings.

## Results

### Metrological information

In the first experiment of the winter season, the average values of the ambient temperature (AT, °C), relative humidity (RH %), and temperature humidity index (THI) were 20.86 ± 0.58, 63.18 ± 1.16, and 20.12, respectively. In the summer season, the equivalent figures for the second trial were 30.90 ± 0.45, 81.63 ± 0.93, and 29.96. Pregnant female rabbits in the winter are subjected to no heat stress, but animals in the summer are exposed to severe heat stress, according to Marai et al. (2001) definition of THI thresholds for heat stress. The findings showed that, the pregnant female rabbits are exposed to extreme heat stress in the summer and are not in the winter (Table 1).

**Table 1:** Air temperature, relative humidity, Temperature-Humidity Index during the two experimental periods inside the Rabbitry building

Experimental periods	Experimental months	Temperature (^o^ C)	Relative humidity (%)	Temperature humidity index (THI)
First experiment (winter season)	January 2023	20.28 ± 0.17	63.33 ± 1.03	19.61
	February 2023	21.43 ± 0.25	63.03 ± 1.47	20.62
	Mean ± SE	20.86 ± 0.58	63.18 ± 1.16	20.12 absence of heat stress
Second experiment (summer season)	July 2023	31.35 ± 0.39	81.05 ± 0.82	30.9
	August 2023	30.45 ± 0.43	82.20 ± 1.79	29.6
	Mean ± SE	30.90 ± 0.45	81.63 ± 0.93	29.96 severe heat stress

The combined effect of the ambient air temperature and relative humidity as temperature humidity index (THI) was calculated using the equation of Marai et al. (2001) as follows: THI = db ^o^C – [(0.31–0.31 RH %) (db ^o^C −14.4)], where db °C = dry bulb temperature in Celsius and RH = relative humidity percentage/100 and the obtained THI values were classified as follow: < 27.8 = absence of heat stress, 27.8 to < 28.9 = moderate heat stress, 28.9 to < 30.0 = severe heat stress and 30.0 and more = very severe heat stress

### Effects of heat stress and Bio-C Plus on respiration rate, ear temperature, body weight and feed intake in pregnant rabbits

Pregnant rabbits exposed to a hot summer had considerably greater (*P* < 0.001) respiration rates (100.36 vs. 78.76) and ear temperatures (38.72 vs. 37.26 °C) than rabbits subjected to a winter without heat stress. The addition of Bio-C Plus to the drinking water of pregnant rabbits exposed to the hot summer months resulted in a substantial (*P* < 0.05) reduction in both respiration rate and ear temperature. The respiration rate decreased from 100.36 to 87.80 rpm and the ear temperature dropped from 38.72 °C to 37.90 °C (Table 2).

**Table 2:** Effect of thermal stress and Bio-C plus on doe respiration rate (RR), ear temperature (ET), doe body weight (BW) and daily feed intake (DFI) of pregnant rabbits during the two experimental periods

RR (rpm), ET (°C), BW and FI	1st experiment (Winter season)	2nd experiment (Summer season)	
		Without treatment	With Bio-C plus
Average of RR, rpm	78.76^c^ ± 1.64	100.36^a^ ± 3.50	87.80^b^ ± 1.77
Change % & Significant		+ 27.44 (*P* < 0.01)	+ 11.48 (*P* < 0.05)
Average of ET	37.26^c^ ± 0.08	38.72^a^ ± 0.06	37.9^b^ ± 0.05
Change & Significant		+ 1.46 °C(*P* < 0.01)	+ 0.64 °C(*P* < 0.05)
Average of doe body weight, g	3985.7^a^ ± 43	3850.7^c^ ± 68	3919.8^b^ ± 47
Change & Significant		−135.0 g (*P* < 0.01)	−65.9 g (*P* < 0.05)
Increase due to Bio-C Plus			+ 69.1 g day
Average of doe daily FI, g	213.0^a^ ± 24.3	171.4^c^ ± 8.8	193.8^b^ ± 16.7
Change & Significant		−41.6 g (*P* < 0.01)	−19.2 g (P < 0.05)
Increase due to Bio-C Plus			+ 22.4 g /day

a, b…Means in the same row within each item having different superscripts differ significantly at *p* < 0.05

Live body weight and feed intake decreased dramatically (P < 0.01) when pregnant rabbits were subjected to extreme heat stress in the summer in comparison to live body weight and feed intake in pregnant rabbits exposed to the winter season. Both the total mean body weight and the total mean daily feed consumption dropped by 135.00 g and 41.69 g/day, respectively. When Bio-C Plus was added to the drinking water of pregnant rabbits exposed to the summer heat, the drop in body weight and daily feed consumption were reduced. The decline in body weight was lessened to 65.9 g/day and the daily feed intake was reduced to 19.2 g/day (Table 2).

### Effects of heat stress and Bio-C Plus on conception rate of doe rabbits

When rabbits are exposed to extreme heat stress during the summer, the number of pregnant rabbits drops dramatically from 20 in the winter season group to 12 in the hot summer season group. The female rabbits in the winter group had a conception rate of 100%, but the group under temperature stress saw a substantial fall in CR to 60% (- 40%; *P* < 0.01) (Table 3).

**Table 3:** Effect of thermal stress and Bio-C plus on conception rate (CR) of doe rabbits

Conception rate	1st experiment (Winter season)	2nd experiment (Summer season)	
		Without treatment	With Bio-C plus
Number of does at mating	20	20	20
Number of pregnant doe	20.0	12.0	18.0
Conception rate, %	100^a^	60^c^	90^b^
Conception rate change %		−40.0 (P < 0.01)	−10.0 (*P* < 0.01)
Improvement % due to Bio-C plus			+ 50.0 (*P* < 0.001)

a, b…Means in the same row within each item having different superscripts differ significantly at *p* < 0.05

By adding Bio-C Plus to the drinking water of pregnant rabbits exposed to the hot summer months, the decline in conception rate was reduced. The conception rate increased (P < 0.01) from 60% in the group without Bio-C Plus to 90% in the group with Bio-C Plus. The improvement in conception rate due to Bio-C plus was 50% (Table 3).

### Effects of heat stress and Bio-C Plus on progesterone, γ-globulin and antioxidants

Table 4 present that progesterone levels dramatically dropped from 5.91 ng/ml in animals of the winter group to 3.13 ng/ml (−47.04%; *P* < 0.01) in pregnant rabbits exposed to the hot summer months. When pregnant rabbits were given Bio-C Plus to their drinking water throughout the summer, progesterone levels increased to 4.92 ng/ml (+ 16.75% *P* < 0.05). The improvement in progesterone levels due to Bio-C plus was 1.79 ng/ml (57.19%)..

**Table 4:** Effect of thermal stress and Bio-C plus on Progesterone, γ- globulin and antioxidants parameters in pregnant doe rabbits before kindling

Blood analysis	1st experiment (Winter season)	2nd experiment (Summer season)	
		Without treatment	With Bio-C plus
Progesterone hormonal level (ng/ml)	5.91^a^ ± 0.40	3.13^c^ ± 0.37	4.92^b^ ± 0.57
Change % & Significant		−47.04(*P* < 0.01)	−16.75(*P* < 0.05)
Improved due to Bio-C plus			1.79 ng/ml (57.19%)
γ- globulin (g/dl)	1.73^a^ ± 0.03	1.19^c^ ± 0.03	1.47^b^ ± 0.05
Change % & Significant		−31.21(*P* < 0.01)	−15.03(*P* < 0.05)
Improved due to Bio-C plus			0.28 g/dl (23.53%)
Glutathione peroxidase, μmol/dl	27.65^a^ ± 1.54	22.60^c^ ± 0.21	25.37^b^ ± 1.07
Change % & Significant		−18.26 (*P* < 0.05)	−8.25 (*P* < 0.05)
Improved due to Bio-C plus			3.31 μmol/dl (14.60%)
Superoxide dismutase, U/dl	9.00^a^ ± 0.96	7.42^c^ ± 0.16	8.26^b^ ± 0.63
Change % & Significant		−17.56 (*P* < 0.05)	−8.22 (*P* < 0.05)
Improved due to Bio-C plus			0.84 U/dl (11.32%)

a, b…Means in the same row within each item having different superscripts differ significantly at *p* < 0.05

Pregnant rabbits had a gamma globulin concentration of 1.73 mg/dl in the winter group, which dropped dramatically (−31.21%; *P* < 0.01) to 1.19 mg/dl in the group of the hot summer months. Adding Bio-C Plus to their drinking water throughout the summer, the concentration rose to 1.47 mg/dl (15.03%). The improvement in gamma globulin concentration due to Bio-C plus was 0.28 g/dl (23.53%) as presented in Table (4).

The activity of glutathione peroxidase and superoxide dismutase showed a similar pattern. The activities of GSH and SOD were significantly lower in animals exposed to severe heat stress by 18.26 and 17.26%; *P* < 0.05, respectively, compared to rabbits exposed to the absence of heat stress. Adding Bio-C Plus to the drinking water of pregnant rabbits exposed to the hot summer months, the activities were greatly restored to most of their previous levels. GSH and SOD activity were considerably higher than those of the group that did not receive Bio-C Plus in the drinking water. The improvement in GSH and SOD activities due to Bio-C plus were 3.31 μmol/dl (14.60%) and 0.84 U/dl (11.32%), respectively (Table 4).

### Effects of heat stress and Bio-C Plus on doe milk yield of rabbits after kindling

The average milk yield of the rabbits during their weeks of nursing was 181.63 ml overall in the winter, but this amount plummeted substantially to 139.91 ml (−22.97%; *P* < 0.01) when they were subjected to the hot summer months. When Bio-C Plus was added to the drinking water of pregnant rabbits during the summer, the mean amount of milk produced by the rabbits increased to 162.08 ml (+ 15.85%;*P* < 0.05). The second week had the highest milk output, while the first and fourth weeks had the lowest, respectively (Table 5).

**Table 5:** Effect of thermal stress and Bio-C plus on doe milk yield of doe rabbits after kindling from mating till day 28

Milk yield, g/day	1st experiment (Winter season)	2nd experiment(Summer season)		Overall mean due to week
		Without treatment	With Bio-C plus	
Average milk yield at day 7	167.20^a^ ± 3.4	113.80^c^ ± 3.8	139.40^b^ ± 3.5	140.13^C^ ± 3.4
Average milk yield at day 14	236.10^a^ ± 4.3	188.60^c^ ± 4.2	212.10^b^ ± 4.3	212.27^A^ ± 4.6
Average milk yield at day 21	184.30^a^ ± 4.1	155.60^c^ ± 4.1	174.50^b^ ± 4.1	171.47^B^ ± 3.6
Average milk yield at day 28	138.90^a^ ± 3.0	101.65^b^ ± 2.3	122.30^c^ ± 3.5	120.95^D^ ± 2.6
Overall mean of daily milk yield	181.63^a^ ± 20	139.91^c^ ± 19.9	162.08^b^ ± 19.9	161.21 ± 20.0
Change %		−22.97(*P* < 0.01)	−10.76(*P* < 0.5)	
Improvement due to treatment			+ 15.85(*P *< 0.5)	

a, b…Means in the same row within each item having different superscripts differ significantly at *p* < 0.05

A, B…Means in the same column having different superscripts differ significantly at *p* < 0.05

### Effects of heat stress and Bio-C Plus on the development of the young rabbits

The number of bunnies born in a group of rabbits subjected to winter was 130.0. In the group exposed to extreme heat stress, the number of bunnies born dropped to 70.00 babies. The number of bunnies born in the group of rabbits subjected to drinking water infused with Bio-C Plus during the sweltering summer improved to 110 bunnies. The equivalent figures at weaning were 124.0, 55.0, and 100.0. The number of bunnies at birth for each doe in the group subjected to winter was 6.5 bunnies and decreased to only 5.83 bunnies in the group exposed to the summer. In the group of rabbits drinking Bio-C Plus water during the sweltering summer increased to 6.11 bunnies for each doe. The equivalent figures at weaning were 6.2, 4.58, and 5.56. Bunnies subjected to winter had an average weight of 59.4 g at birth, whereas those exposed to extreme heat stress during the summer had an average weight of 48.9 g.

During the sweltering summer, the average weight of the rabbits in the group that drank water infused with Bio-C Plus was 53.5 g. At weaning, the average weight of the bunnies was 595.9, 478.4, and 530.0 g, respectively. The total bunny weight at birth for each doe in the group exposed to winter was 386.1 g, while the average litter weight in the group subjected to extreme heat stress during the hot summer months dropped to 285.08 g. The total rabbits weight for each doe of the rabbits in the hot summer group that drank water infused with Bio-C Plus at birth increased to 326.89 g. At weaning, the total bunny weight for each doe was 3694.58, 2191.07 and 2946.80 g, respectively (Table 6).

**Table 6:** Effect of thermal stress and Bio-C plus on young rabbits from birth to weaning

Young rabbits from birth to weaning	1st experiment (Winter season)	2nd experiment (Summer season)	
		Without treatment	With Bio-C plus
Number of does at kindling	20	12	18
At birth			
Number of babies born in the group	130.0	70.0	110.0
Number of babies born in each doe	6.50^a^ ± 0.18	5.83^c^ ± 0.11	6.11^b^ ± 0.14
Average of bunny weight in each doe (g)	59.4^a^ ± 1.19	48.9^c^ ± 1.44	53.5^b^ ± 1.3
Total bunny weight in each doe (g)	386.1^a^ ± 10.0	285.08^c^ ± 9.4	326.89^b^ ± 7.6
Total bunny weight in the group (kg)	50.19^a^ ± 50,3	19.96^c^ ± 30.4	35.96^b^ ± 34.5
At weaning			
Number of babies in the group	124	55.0	100.0
Number of babies born in each doe	6.20 ^a^ ± 0.18	4.58 ^c^ ± 0.17	5.56 ^b^ ± 0.11
Average of bunny weight in each doe (g)	595.9^a^ ± 14.1	478.4^c^ ± 11.2	530.0^b^ ± 10.2
Total bunny weight in each doe (g)	3694.58^a^ ± 20.5	2191.07^c^ ± 12.5	2946.80^b^ ± 18.7
Total bunny weight in each group (kg)	73.89^a^ ± 126	26.31^c^ ± 96	53.00^b^ ± 110
Change % in bunny weight in the group		−47.58 kg 64.39(P < 0.01)	−20.89 kg 28.27(P < 0.01)
Improvement due to Bio-C, kg			+ 26.69 kg (P < 0.01)
Mortality from birthing to weaning, No (%)	6 0.0 (4.61)	15.0 (21.43)	10.0 (9.09)
Bunny gain from birthing to day 28 (g/day)	19.16^a^ ± 0.16	15.34^c^ ± 0.22	17.02^b^ ± 0.13
Change % in bunny weight gain		−19.94(P < 0.05)	−11.17(P < 0.05)
Improvement % in bunny gain due to Bio-C			+ 11.0 (P < 0.05)

Average number of babies born in each doe at birth or at weaning = Number of babies born in the group / Number of does at kindling

Average bunny weight in each doe = Number of babies born in each doe X Bunny weight in each doe

Average bunny weight in each group = Number of babies born in each group X Bunny weight in each group

Mortality from birthing to weaning = Number of babies born in the group at weaning—Number of babies born in the group at birth

Bunny gain from birthing to day 28 (g/day) = (bunny weight at weaning—bunny weight at birth) /28

a, b…Means in the same row within each item having different superscripts differ significantly at *p* < 0.05

The total bunny weight at birth for each group exposed to winter was 50.19 kg and decreased to 19.96 kg in the group subjected to extreme heat stress during the hot summer months. The total rabbit’s weight at birth for each group that drank water infused with Bio-C Plus was 35.96 kg. At weaning, the total weight of the bunnies in each group was 73.89, 26.31, and 53.0 kg, respectively. When compared to rabbits who did not receive Bio-C Plus in their drinking water during the hot summer, the total bunny weight at weaning improved 20.89 kg (28.89%; *P* < 0.01). However, the summer heat stress dropped the winter group's total bunny weight by 47.58 kg (64.39%, *P* < 0.01). The group that received Bio-C Plus in the drinking water during the summer noticed a drop in total bunny weight from 47.58 kg in the winter to 20.89 kg (28.27%, *P* < 0.01). In comparison to the group that did not receive Bio-C Plus, the group that obtained Bio-C throughout the summer had an improvement in total bunny weight of 26.69 kg (*P* < 0.01). From birth until weaning, the mortality rates were 6.0 (4.61%), 15.0 (21.43%), and 10.0 (9.09%) in that order. Thus, the vitality percentage from birthing to weaning in the three experimental groups was 95.39, 78.57, and 90.91%, respectively. The weight increase of the bunnies from birth to day 28 of the nursing period was 19.16 g in the group exposed to winter, and it dropped dramatically to 15.34 g (−19.94; *P* < 0.05) in the group subjected to extreme heat stress during the hot summer months. When compared to the group of rabbits that did not get Bio-C Plus in drinking water throughout the hot summer, the average weight of the bunnies from birth to day 28 of the nursing period improved to 17.02 g (+ 11.0; *P* < 0.05) in the Bio-C Plus-received group (Table 6).

## Discussion

### Effects of heat stress and Bio-C Plus on respiration rate and ear temperature

The current results revealed that rabbits under thermal stress had significantly higher ear temperatures and respiration rates than rabbits under non-heat stress. Adding Bio-C Plus to drinking water throughout the hot summer months significantly (*P* < 0.05) decreased both respiration rate and skin temperature.

In rabbits, the majority of sweat glands are inactive and do not produce sweat. Rabbits can only regulate their breathing rate to remove extra body heat through thermoregulation (Rafel et al. 2012). Since pulmonary ventilation plays a major role in regulating a rabbit's body temperature in hot weather, an increase in respiration rate may aid in increasing heat loss via pulmonary ventilation processes. Animals that drink a lot of water during the summer months lose more heat by vaporizing the water in their respiratory systems (Badr 2015). Rabbits, as homoeothermic animals, may use thermoregulatory processes to control the amount of heat that enters and leaves their bodies to maintain a somewhat normal body temperature (Habeeb 2020). When a rabbit is subjected to heat stress, their ear temperatures rise because the rabbit's skin acts as a radiator, boosting heat loss from the ear (Sabah et al. 2016). High concentrations of ascorbic acid (vitamin C; 180 g/kg of Bio-C Plus), citric acid and vitamin E included in Bio-C Plus functions as an antioxidant may help the heat-stressed rabbits breathe easier and feel cooler on the skin. Similarly, male rabbits exposed to a hot summer and received vitamins AD3E had less negative heat stress on their physiological thermoregulatory parameters (Sharaf et al. 2019).

### Effects of heat stress and Bio-C Plus on body weight and feed intake of doe

The live body weight and feed intake of pregnant rabbits decreased dramatically (P < 0.01) due to being subjected to the hot summer months in comparison to the rabbits exposed to winter months. Heat stress causes significant changes in rabbits' biological functions, which causes their body weight and feed intake to drop (Habeeb et al. 2023). The observed decrease in body weight might be the result of a lower feed intake, which lowers the rate of metabolism, lessens fat deposition, inhibits the production of digestive enzymes, and reduces protein synthesis (Ogunjimi et al. 2008). The decrease in body weight might perhaps be attributed to a rise in energy dissipation during macromolecule synthesis, with the majority of energy being used for the body's thermoregulatory processes through elevated respiration rates (Garlick 2005).

The observed drop in feed intake may be the consequence of high temperatures stimulating peripheral thermal receptors, which transmit inhibitory nerve signals to the hypothalamic appetite center, causing the rabbits to consume less feed (Habeeb et al. 1992). Rabbits may eat less feed during the hot summer months in order to minimize the quantity of heat created by metabolism for physiological and biochemical changes (Li et al. 2020).

The drop in body weight and daily feed consumption in heat-stressed rabbits improved significantly (P < 0.05) due to adding Bio-C Plus to the drinking water rabbits. Doe daily body weight rose 69.1 g, and daily feed consumption of each doe increased 22.4 g. It seems that the inclusion of Bio-C Plus, vitamins C and E, and a few trace minerals helped to mitigate the considerable changes in the biological function of rabbits that cause a fall in body growth and feed intake. Animals are not able to manufacture vitamin E, a powerful antioxidant, in their bodies. Vitamin E in Bio-C Plus is important for antioxidant activity and activation of the immune system (Hafez 2012).

### Effects of heat stress and Bio-C Plus on the conception rate of rabbits

The number of pregnant rabbits rapidly drops from twenty (100%) in the winter season group to twelve (60%) in the hot summer season group. Adding Bio-C Plus to the drinking water of a heat-stress doe increased the rate of conception to 90%.

The heat stress of the summer months severely impacts the biological function of rabbits, which lowers the rate of conception. Most females reject males approaching them, female rabbits' urge to mate also wanes throughout the sweltering summer months (Marai et al. 2002). Additionally, in situations of heat burden, a portion of the sperm during mating may be absorbed in the uterine root, hence decreasing the rate of conception. The negative effects reduction in daily feed intake of doe exposure to heat stress conditions reduced ovulation and decreased rates of conception due to inadequate or unbalanced nutrient intake (Habeeb et al. 2013).

Bio-C Plus contains 30 g of zinc sulfate per kg. More than 200 enzymes include zinc, which also serves as a cofactor in more than 200 enzymes and boosts the activity of around 100 other enzyme components that support an animal's metabolic processes. When animals received zinc in their food, their conception rate tended to improve (Bosseboeuf et al. 2006). This improvement may be due to the fact that zinc is essential for ovulation and fertilization (Yanagisawa 2008).

### Effects of heat stress and Bio-C Plus on progesterone, γ- globulin and antioxidants parameters

Pregnant rabbits exposed to heat stress had significant drops in blood progesterone and γ-globulin concentrations, as well as in the activities of superoxide dismutase and glutathione peroxidase. After adding Bio-C Plus to their drinking water, pregnant rabbits subjected to the hot summer months showed significant restoration of most of their progesterone levels, serum γ-globulin concentrations, and glutathione peroxidase and superoxide dismutase activity.

Elevated ambient temperature has an impact on peripheral thermal sensors in any animal, which relay nerve impulses to the pituitary gland, hypothalamus, and sex gland, ultimately lowering the progesterone level**.** The addition of Bio-C Plus may have increased the progesterone levels in the rabbits, perhaps improving general health and reducing heat stress in the pregnant rabbits (Habeeb 2019). Zinc affects progesterone hormones, which include sharing prolactin and influencing the excretion of prostaglandins, androgens, and gonadotrophins. Zinc also acts as an activator of enzymes required for steroidogenesis, which controls gonadal hormone release (Sharma and Joshi 2005).

The observed drop in serum γ-globulin content in heat-stressed rabbits may be due to the reduction in feed intake in this study. Zinc in Bio-C Plus contributes to the mechanisms of the main metabolic pathways involving protein synthesis and turnover of carbohydrates, energy, nucleic acids, lipids, and heme syntheses involved in the animal immune system, as demonstrated by an increase in the concentration of γ-globulin (Babaei et al. 2007).

Oxidative damage frequently follows heat-stress and impairs an animal's ability to function. SOD and GSH activity decreased in the doe exposed to heat stress conditions in our study; consequently, oxidative damage will increase in the heat-stressed doe. It is well known that GSH activity plays a significant part in the oxidative defense by reducing hydrogen and lipid peroxides in rabbit tissues. While SOD plays a significant part in catalyze the conversion of superoxide to hydrogen peroxide (H_2_O_2_) into oxygen and water (Mutwedu et al. 2021). Vitamin E in Bio-C Plus scavenges reactive oxygen species (molecules that cause harm to cells) and breaks oxidative chain reactions, limiting lipid peroxidation (Wolfs 2005). Vitamin E in Bio-C Plus also inhibits the propagation of free radicals by reacting with them to produce the tocopheryl radical, which is subsequently reduced and returned to its initial state by a hydrogen donor (Moreira et al. 2010).

### Effects of heat stress and Bio-C Plus on the doe milk yield

The rabbits were nursed during the hot summer months, resulting in a 22.97% drop in their average milk output. The lower feed intake seen in this study and the whole mammary gland in the heat-stressed rabbits may have a detrimental effect on milk synthesis in the udder and, consequently, on the output of milk in the breastfeeding rabbits. The findings of our investigation demonstrated that the physiological performance of female rabbits under heat stress was enhanced when Bio-C Plus was added to their drinking water. This enhancement may result in higher feed intake and metabolic rates, which would raise the milk production of pregnant rabbits by 15.8%. In all experimental groups, the maximum milk supply occurred in the second week and the lowest in the fourth. Potential causes of the variations in doe milk supply throughout the suckling phase might include the different sizes of litters in each experimental group.

### Effects of heat stress and Bio-C Plus on the offspring performance

Rabbits exposed to the hot summer throughout their pregnancy produced smaller litter overall, average doe litter sizes at birth, and lower total litter weights at birth after kidding. The preceding measures showed a similar pattern during weaning. From birth until weaning, the mortality rates increased in heat-stressed rabbits and improved due to Bio-C Plus adding**.** The bunny daily gain of mothers exposed during the pregnancy period to the hot summer season decreased and improved due to Bio-C Plus adding. When the temperature outside rises over the rabbits their thermal comfort zone, their productivity is reduced. Reduced daily feed intake and, as a result, poorer milk production from rabbits exposed to the hot summer months may be the cause of the fall in these parameters in offspring from birth to weaning. The high temperature may have harmed their incapacity to produce milk, as well as the growth and well-being of the kits and the mother's ability to take in food. The performance of the progeny increased in those given Bio-C Plus in their drinking water during the scorching summer. This enhancement could be the result of increasing the amount of milk supply to the bunnies.

It can be concluded that exposure of pregnant female rabbits to hot summer weather decreased the doe weight, feed intake, and conception rate, as well as the levels of antioxidant markers. The doe's milk production throughout the nursing period and the growth of the progeny from birth to weaning are negatively impacted by the hot summer months. When the supplement Bio-C Plus was added to the drinking water of pregnant female rabbits, the majority of these degrading effects were reduced.

## Data Availability

All authors confirmed that the availability of data and materials support their published claims and comply with field standards.

## Code availability

All authors confirmed that the availability of software application or custom code supports their published claims and comply with field standards.
